# Research on Bacterial Virulence in the Developing Countries

**DOI:** 10.1155/2015/972187

**Published:** 2015-02-02

**Authors:** Angel Cataldi, Ana Lucia Nascimento, Armando Acosta, Chensong Wan

**Affiliations:** ^1^Institute of Biotechnology, INTA, Hurlingham, Argentina; ^2^Centro de Biotecnologia, Instituto Butantan, Sao Paulo, Brazil; ^3^Finlay Institute, Havana, Cuba; ^4^Southern Medical University, Guangzhou, Guangdong, China

This special issue deals with the advances in the investigation of bacterial virulence in developing countries.

Developing countries, underdeveloped countries, Third World countries, and Southern countries are all definitions used in the academic world to design less industrialized countries with lower income. Even though these terms are ambiguous, we mostly have a clear idea of what we are talking about when we say “developing countries.”

In those countries, there is currently a fervent scientific research activity. An example is the case of microbiology. This science has a long standing history in developing countries. The clear evidence of smallpox vaccination in China in 1000 and 1600 under the rule of Emperor Káng (Ian Glynn and Jenifer Glynn, The Life and Death of Smallpox). The discovery of the cholera toxin in Calcutta 1959 by Sambhu Nath De. The Cuban researcher Carlos Finlay proved that* Aedes aegypti* carries the agent of yellow fever. The work of Carlos Chagas in Brazil showed that a parasite was the agent in Chagas disease. These are all brilliant examples of microbiology research in southern countries. Those scientists had the merit and the bravery of not merely providing samples and field cases so that other scientists with more resources could continue their work carrying out the research themselves, there, on the battle field, despite harsh conditions and budgetary restrictions. That pioneer research continues today, even when conditions are detrimental to its growth.

The objective of this special issue is to give visibility to the research into topic of bacterial virulence by bacteria pathogenic to human and animals. The contributions published in this issue aim to reflect the diversity of research on bacterial virulence in southern countries as follows:

- transporter or translocation mechanisms related to virulence in* Mycobacterium avium *subsp*. avium *(M. N. Viale et al.),* Corynebacterium pseudotuberculosis* (P. M. R. Oliveira Moraes et al.), and enteropathogenic* E. coli* (S. C. F. Sampaio et al.);
- toxins in* Staphylococcus aureus* isolated in Nepal (B. Shrestha et al.) and in enteropathogenic* E. coli* (R. Cássia Ruiz et al.);
- antigens as immunogens for vaccination against* Bordetella pertussis *(N. Olivera et al.),* M. tuberculosis* (L. Novoa-Aponte and C. Y. Soto Ospina), and* Leptospira *(M. V. Atzingen et al.) or dedicated to immunodiagnosis in veterinary diseases (M. L. Mon et al.) or to specific strains of* M. tuberculosi*s (P. Schierloh et al.);
- adhesins in* Mycobacterium avium* subsp.* paratuberculosis* (M. N. Viale et al.);
- the repertoire of virulence factors in* Shigella* from Amazonian children (M. C. Scheffer de Souza et al.), in* Escherichia coli* isolates from women with urinary tract infection in Mexico (D. A. López-Banda et al.), and in retail meat (P. Llorente et al.);
- genomic, comparative genomic, and phylogenetic studies of* Vibrio parahaemolyticus* Hemolysin (S. K. Bhowmik et al.),* Stenotrophomonas* spp. (V. Godoy Cerezer et al.), and* Helicobacter pylori *(Ali et al.);
- characterization of enterotoxigenic* Escherichia coli *in Colombia (J. A. Guerra et al.);
- physiopathology of Shiga toxin in pregnancy (F. Sacerdoti et al.);
- virulence regulation by smoke in* Streptococcus pneumoniae* (R. Cockeran et al.);
- native immune response to* Staphylococcus aureus* (N. Alva-Murillo et al.) and* M. tuberculosis* (A. C. Helguera-Repetto et al.), and biofilm formation (H. F. Culler et al.);
- a model of pathogenic* E. coli *in ameba (F. Yousuf et al.).

*Angel Cataldi* *Ana Lucia Nascimento* *Armando Acosta* *Chensong*  *Wan*

